# Target volume delineation for radiotherapy of meningiomas: an ANOCEF consensus guideline

**DOI:** 10.1186/s13014-023-02300-w

**Published:** 2023-07-05

**Authors:** Nicolas Martz, Julia Salleron, Frédéric Dhermain, Guillaume Vogin, Jean-François Daisne, Raphaelle Mouttet-Audouard, Ronan Tanguy, Georges Noel, Matthieu Peyre, Isabelle Lecouillard, Julian Jacob, Justine Attal, Marie Charissoux, Ovidiu Veresezan, Chantal Hanzen, Aymeri Huchet, Igor Latorzeff, Alexandre Coutte, Jérôme Doyen, Dinu Stefan, Loic Feuvret, Gabriel C. T. E. Garcia, Philippe Royer

**Affiliations:** 1 Academic Department of Radiation Therapy & Brachytherapy, Institut de Cancérologie de Lorraine - Alexis-Vautrin Cancer Center, 6 avenue de Bourgogne – CS 30 519, Vandoeuvre Les Nancy, France; 2 Cellule Data-biostatistiques, Institut de Cancérologie de Lorraine, Université de Lorraine, Vandœuvre-lés-Nancy, France; 3 Department of Radiation Oncology, Gustave Roussy University Hospital, Villejuif, France; 4 Department of Radiation Therapy, Baclesse Radiation Therapy Centre, Esch/Alzette, Luxembourg; 5 Department of Radiation Oncology, University Hospitals Leuven, Catholic University of Leuven (KU Leuven), Leuven, Belgium; 6 Academic Department of Radiotherapy, Oscar Lambret Comprehensive Cancer Centre, Lille, France; 7 Department of Radiotherapy, Léon Bérard Cancer Centre, Lyon, France; 8 Radiation Oncology Department, Paul Strauss Cancer Centre, Strasbourg, France; 9 Department of Neurosurgery, Sorbonne Université, Groupe Hospitalier Pitié-Salpêtrière, Paris, France; 10 Department of Radiation Oncology, Eugène Marquis Cancer Centre, Rennes, France; 11 Department of Radiation Oncology, Hôpital Pitié-Salpêtrière Charles Foix, APHP, Paris, France; 12 Radiotherapy Department, Institut Claudius Regaud, Toulouse University Institute for Cancer (IUCT-Oncopole), Toulouse, France; 13 Radiation Oncology Department, Centre Val d’Aurelle, Montpellier, France; 14 Department of Radiation Oncology, Cancer Centre Henri Becquerel, Rouen, France; 15 Department of Radiation Oncology, Centre Hospitalier et Universitaire, Bordeaux, France; 16 Department of Radiotherapy, Groupe ONCORAD Garonne and Clinique Pasteur, Toulouse, France; 17 Department of Radiation Therapy, CHU d’Amiens, Amiens, France; 18 Department of Radiation Therapy, Antoine Lacassagne Cancer Center, University of Nice- Sophia, Nice, France; 19 Department of Radiation Oncology, François Baclesse Cancer Centre, Caen, France; 20 Department of Diagnostic Radiology, Gustave Roussy, Villejuif, F-94800 France

**Keywords:** Meningiomas, Radiotherapy, Delineation, Target volume, Consensus guidelines

## Abstract

**Purpose:**

Radiotherapy is, with surgery, one of the main therapeutic treatment strategies for meningiomas. No prospective study has defined a consensus for the delineation of target volumes for meningioma radiotherapy. Therefore, target volume definition is mainly based on information from retrospective studies that include heterogeneous patient populations. The aim is to describe delineation guidelines for meningioma radiotherapy as an adjuvant or definitive treatment with intensity-modulated radiation therapy and stereotactic radiation therapy techniques. This guideline is based on a consensus endorsed by a multidisciplinary group of brain tumor experts, members of the Association of French-speaking Neuro-oncologists (ANOCEF).

**Materials and methods:**

A 3-step procedure was used. First, the steering group carried out a comprehensive review to identify divergent issues on meningiomas target volume delineation. Second, an 84-item web-questionnaire has been developed to precisely define meningioma target volume delineation in the most common clinical situations. Third, experts members of the ANOCEF were requested to answer. The first two rounds were completed online. A third round was carried out by videoconference to allow experts to debate and discuss the remaining uncertain questions. All questions remained in a consensus.

**Results:**

Limits of the target volume were defined using visible landmarks on computed tomography and magnetic resonance imaging, considering the pathways of tumor extension. The purpose was to develop clear and precise recommendations on meningiomas target volumes.

**Conclusion:**

New recommendations for meningiomas delineation based on simple anatomic boundaries are proposed by the ANOCEF. Improvement in uniformity in target volume definition is expected.

**Supplementary Information:**

The online version contains supplementary material available at 10.1186/s13014-023-02300-w.

## Introduction

Meningiomas are the most common primary intracranial tumors in adults, representing 36.8% of primary central nervous system (CNS) tumors [1]. The overall incidence almost doubled from 4.52 to 8.3 per 100,000 people between 1998 and 2002 and 2010–2014 [1, 2]. It has not been established whether this increase is real or related to more frequent incidental detection by neuroimaging. According to the World Health Organization (WHO) classification, 81.3% of meningiomas are grade I (typical), 16.9% are grade II (atypical), and 1.7% are grade III (anaplastic) [1].

Definitive radiotherapy (RT) is reserved for symptomatic, progressive, and/or life-threatening inoperable meningiomas. Postoperative RT (poRT) is discussed for grade II and indicated for grade III meningiomas to improve local control. Moreover, stereotactic radiation therapy (SRT) has gradually emerged as an alternative for patients with small volume, recurrent, or high-risk surgical meningiomas, mostly those located at the skull base [3, 4].

Improvements in RT techniques have led to a progressive decrease in planning target volume margins (PTV). At the same time, the evolution of diagnostic imaging has allowed more precise definition of gross and clinical target volumes (GTV/CTV) and raises new questions about clinically relevant tumor extension. However, no randomized trial has examined the impact of decreased margins on local control rates. These margins must be adapted to the risk of local recurrence, which differs depending on the meningioma grade [5]. Current recommendations are based on European Society for Radiation and Oncology Advisory Committee on Radiation Oncology Practice (ESTRO-ACROP) and National Comprehensive Cancer Network (NCCN) guidelines which provide few details on target volume delineation and additional (CTV) margins [6, 7].

Our multidisciplinary initiative aims to define delineation guidelines for the GTV and CTV of meningiomas as adjuvant or definitive treatment. Our manuscript aims to define the CTV as a clinically relevant volume that achieves satisfactory local control while limiting the risks of RT-related complications in these long-surviving patients.

## Materials and methods

A multidisciplinary group of expert radiation oncologists, neuroradiologists, and neurosurgeons was recruited through the mailing list of the Association of French-speaking Neuro-oncologists (ANOCEF). Expert centers were defined as those managing a least 20 meningiomas per year.

First, the steering group, developed through a muldisciplinary approach (3 radiation oncologists, 1 radiologist and 1 statistician), carried out a comprehensive review. A search of MESH terms including “meningioma”, “radiotherapy”, and “target volume” allowed us to compare the different delineation methods described in the studies and identify controversies. The steering group developed a web questionnaire with 84 questions, designed in two parts: one on intensity-modulated radiation therapy (IMRT) and the other on SRT (Appendix 1). Each part was divided into subsections about imaging modalities necessary for delineation, and target volumes (GTV/ CTV) sorted by grade. Each question was scored by experts from 1 (strongly disagree) to 9 (totally agree) during a two-round modified Delphi process [8, 9]. The experts had three weeks to answer each round. Between rounds, experts received an automatically generated personalized report showing their individual responses compared with the responses of other experts. Questions that did not reach a consensus in the first round were submitted to the second round, to score them in light of the responses (quantitative feedback) and arguments (qualitative feedback) of all co-authors. Some questions were reformulated or clarified between rounds. During these two rounds, experts were not allowed to communicate, to avoid bias.

The consensus was adopted by the RAND/UCLA appropriateness method [10]. Consensus was reached if the question was rated as “appropriate” or “inappropriate”. If it was rated as uncertain, it did not reach consensus and was included in the second round. A question was judged appropriate when the median value was ≥ 7 and experts agreed; inappropriate when the median value was ≤ 3.5 and experts agreed; and uncertain when the median score was between 4 and 6.5 (indecision) or experts disagreed. Expert disagreement was defined by the value of the interpercentile range adjusted for symmetry (IPRAS) index [10]. For consensus on imaging modalities and margins, a threshold corresponding to a minimum of 80% agreement per question was chosen.

A third round was carried out by videoconference to allow experts to debate and discuss the remaining uncertain questions; this was followed by a vote. The date has been chosen so that most of the experts can participate in one unique session. A question was judged appropriate or inappropriate where there was an absolute majority. If there was not, the question was considered uncertain.

## Results

Twenty-two experts from 18 RT centers in France (15 centers), Belgium (two centers), and Luxembourg (one center) responded favorably. One center was excluded at the time of inclusion because it did not treat the minimum requirement of 20 meningiomas per year. Seventeen centers with 21 experts were included.

The first round of the two-round Delphi process took place in February 2021. The response rate was 95%; one expert was excluded because they did not respond within the time limit. Experts assessed 84 questions for IMRT and SRT by tumor grade. After the first round, experts had reached an agreement on 79% of questions. For the second round, carried out during April 2021, the response rate was 100% (20 experts). Overall, 82% of the questions reached a consensus (79% in IMRT, 89% in SRT). Fifteen questions remained uncertain. After the third round, which took place on November 17, 2021 with 14 experts (70%) participating, all questions had reached a consensus.

### Imaging modality

Regardless of treatment technique, experts considered that an unenhanced planning CT scan combined with postcontrast T1-weighted MRI was mandatory for target volume delineation. For IMRT, fusion with a preoperative MRI for adjuvant RT was at least strongly recommended by 80% of experts (mandatory for 45%, strongly recommended for 35%). Contrast-enhanced planning CT and fusion with a non-contrast-enhanced T1-weighted sequence, a T2-weighted sequence with fluid-attenuated inversion recovery, or a T2-weighted sequence with cancellation of the blood signal by spin echo were optional, according to clinical context. The MRI used for delineation should ideally be performed in the treatment position within 4 weeks of RT, or less for rapidly progressive meningiomas.

### GTV definition

Our experts’ GTV definition, for whole tumor or post-operative residue, included the nodular dural enhancement, contrast-enhancing thickened meninges, and directly invaded bone, regardless of WHO grade and RT technique: IMRT (Table 1) and SRT (Table 2).

**Table 1:** GTV definition for IMRT

Grade	Included structures	RAND/UCLA method			ANOCEF consensus guideline
		1st round	2nd round	3rd round	
I	Nodular enhancement	Appropriate	-	-	Appropriate
	Thickened meninges	Appropriate	-	-	Appropriate
	Directly invaded bone	Appropriate	-	-	Appropriate
II	Nodular enhancement	Appropriate	-	-	Appropriate
	Thickened meninges	Appropriate	-	-	Appropriate
	Directly invaded bone	Appropriate	-	-	Appropriate
III	Nodular enhancement	Appropriate	-	-	Appropriate
	Thickened meninges	Appropriate	-	-	Appropriate
	Directly invaded bone	Appropriate	-	-	Appropriate

ANOCEF, Association of French-speaking Neuro-oncologists; GTV, gross tumor volume; IMRT, intensity-modulated radiation therapy

**Table 2:** GTV and CTV definition for SRT

	Included structures	RAND/UCLA method			ANOCEF consensus guideline
		1st round	2nd round*	3rd round	
GTV	Nodular enhancement	Appropriate	Appropriate	-	Appropriate
	Thickened meninges	Appropriate	Appropriate	-	Appropriate
	Directly invaded bone	Appropriate	Appropriate	-	Appropriate
CTV	Margin around nodular enhancement	Inappropriate	Inappropriate	-	Inappropriate
	Margin along normal meninges	Inappropriate	Inappropriate	-	Inappropriate
	Tumor bed	Uncertain	Uncertain	Inappropriate	Inappropriate
	Margin around tumor bed	Inappropriate	Inappropriate	-	Inappropriate
	Margin in healthy bone	Inappropriate	Inappropriate	-	Inappropriate
	Hyperostosis	Inappropriate	Inappropriate	-	Inappropriate
	Peritumoral edema	Inappropriate	Inappropriate	-	Inappropriate
	Cranial flap	Inappropriate	Inappropriate	-	Inappropriate
	Venous sinus	Inappropriate	Inappropriate	-	Inappropriate
	Other vascular structures (arteries)	Inappropriate	Inappropriate	-	Inappropriate
	Optic nerve	Inappropriate	Inappropriate	-	Inappropriate
	Cranial nerves	Inappropriate	Inappropriate	-	Inappropriate

ANOCEF, Association of French-speaking Neuro-oncologists; CTV, clinical target volume; GTV, gross tumor volume; SRT, stereotactic radiation therapy; * all SRT questions have been clarified and resubmitted for the 2nd round

### IMRT: CTV definition

For IMRT, experts recommended that for grade I tumors, CTVs should correspond to GTVs without additional margins. For grade II tumors, experts recommended adding CTV margins of 5 mm to GTVs in normal brain tissue, hyperostosis, around invaded bone, and along normal (unthickened) meninges and venous sinuses contacting the GTV. For grade III tumors, experts recommended adding CTV margins of 10 mm to GTVs in normal brain tissue, hyperostosis, around invaded bone, and along normal meninges, venous sinuses, and optic or cranial nerves contacting the GTV. Experts recommended that the CTV should include peritumoral oedema only for the first 10 mm around GTV and arterial structures should be excluded (Table 3).

**Table 3:** CTV definition for IMRT

Grade	Included structures	RAND/UCLA method					ANOCEF consensus guideline	Additional margin from GTV (mm)
		1st round	2nd round*			3rd round		
I	Margin around nodular enhancement	Uncertain		Uncertain	Inappropriate		Inappropriate	NA
	Margin along normal meninges	Uncertain		Uncertain	Inappropriate		Inappropriate	NA
	Tumor bed	Uncertain		Appropriate	-		Appropriate	NA
	Margin around tumor bed	Uncertain		Uncertain	Inappropriate		Inappropriate	NA
	Margin in healthy bone	Inappropriate		-	-		Inappropriate	NA
	Hyperostosis	Uncertain		Uncertain	Inappropriate		Inappropriate	NA
	Whole peritumoral edema	Inappropriate		-	-		Inappropriate	NA
	Cranial flap	Inappropriate		-	-		Inappropriate	NA
	Venous sinus	Inappropriate		Inappropriate	-		Inappropriate	NA
	Other vascular structures (arteries)	Inappropriate		Inappropriate	-		Inappropriate	NA
	Optic nerve	Inappropriate		Inappropriate	-		Inappropriate	NA
	Cranial nerves	Inappropriate		Inappropriate	-		Inappropriate	NA
II	Margin around nodular enhancement	Appropriate		-	-		Appropriate	5
	Margin along normal meninges	Appropriate		-	-		Appropriate	5
	Tumor bed	Appropriate		-	-		Appropriate	NA
	Margin around tumor bed	Appropriate		-	-		Appropriate	5
	Margin in healthy bone	Uncertain		Inappropriate	-		Inappropriate	NA
	Hyperostosis	Uncertain		Appropriate	-		Appropriate	5
	Whole peritumoral edema	Inappropriate		-	-		Inappropriate	NA
	Cranial flap	Inappropriate		-	-		Inappropriate	NA
	Venous sinus	Uncertain		Uncertain	Appropriate		Appropriate	5
	Other vascular structures (arteries)	Inappropriate		Inappropriate	-		Inappropriate	NA
	Optic nerve	Inappropriate		Inappropriate	-		Inappropriate	NA
	Cranial nerves	Inappropriate		Inappropriate	-		Inappropriate	NA
III	Margin around nodular enhancement	Appropriate		-	-		Appropriate	10
	Margin along normal meninges	Appropriate		-	-		Appropriate	10
	Tumor bed	Appropriate		Appropriate	-		Appropriate	NA
	Margin around tumor bed	Appropriate		-	-		Appropriate	10
	Margin in healthy bone	Uncertain		Uncertain	Appropriate		Appropriate	10
	Hyperostosis	Appropriate		-	-		Appropriate	10
	Whole peritumoral edema	Inappropriate		-	-		Inappropriate	NA
	Cranial flap	Inappropriate		-	-		Inappropriate	NA
	Venous sinus	Uncertain		Appropriate	-		Appropriate	10
	Other vascular structures (arteries)	Uncertain		Uncertain	Inappropriate		Inappropriate	NA
	Optic nerve	Uncertain		Uncertain	Appropriate		Appropriate	10
	Cranial nerves	Uncertain		Uncertain	Appropriate		Appropriate	10

ANOCEF, Association of French-speaking Neuro-oncologists; CTV, clinical target volume; IMRT, intensity-modulated radiation therapy; NA, not adapted; * all SRT questions have been clarified and resubmitted for the 2nd round

Regardless of tumor grade, cortical bone can be considered an anatomical barrier. Experts recommended that in the absence of invasion, bone should not be included in the CTV.

For poRT, experts made the following additional recommendations: Surgical reports and preoperative and postoperative MRIs are required. Due to the remodeling of the resection cavity, which occurs up to 3 to 5 weeks after surgery, planning imaging should not be performed too early. The tumor bed is defined as the resection cavity (cerebrospinal fluid), with a margin of 1 to 2 mm in brain parenchyma. Additional margins required for tumor bed’s CTV are 0 mm for grade I, 5 mm for grade II, and 10 mm for grade III tumors. The cranial flap should not be included in the CTV (Table 2). For grade II and III tumors, drill holes and osteotomy areas correspond to an area of rupture of the anatomical barrier. They may be included if they come into contact with the CTV. In case of postoperative residue, the additional GTV and CTV is defined as above.

When histology is unavailable, CTV expansion should consider clinical and imaging aspects. Factors raising suspicion of high-grade meningioma are recurrent meningiomas, especially those that recur after surgery and that are not eligible for a second removal, rapidly evolving meningiomas, bone lysis, necrosis, lobulated contours, and a significant brain edema for a moderately sized lesion, which is suggestive of cerebral invasion. In addition, aggressive MRI criteria must be considered: increased perfusion, increased T2 signal intensity, and decreased apparent diffusion coefficients. Meningiomas can be defined as low- or high-grade tumors, and the CTV margin set accordingly, on the basis of concordance of these factors alongside clinical expertise. Diagnosis at a young age (< 60 years), menopausal status, hyperostosis, location, and tumor size were not considered to be relevant factors.

Experts with proton therapy experience agreed that IRMT recommendations are applicable to this technique.

### SRT: CTV definition

For SRT, experts recommended that CTVs should correspond to GTVs without additional margins (Table 2). Experts recommended that in a postoperative situation, only the residual enhancement of the tumor needs to be included and not the excision cavity. Experts did not recommend this technique for grade II and III meningiomas, except in selected cases of recurrence.

## Discussion

Most published studies on meningioma RT are retrospective and do not report sufficient details on target volume delineation and the impact on local control. This article aimed to define delineation recommendations for meningioma RT. Indications/prescribing doses were not addressed.

Imaging modalities considered mandatory by experts are unenhanced planning CT fused with postcontrast T1-weighted MRI. Adding a contrast-enhanced CT has no real added value for delineating target volumes and organs at risk. For patients with MRI contraindications, contrast-enhanced CT is acceptable but provides a lower contrast resolution. However, in skull base meningioma or tumors infiltrating bone, it can be difficult to define the tumor extension on CT images [11, 12]. MRI sequence with cancellation of the blood signal (hypointense) is useful to discriminate meningiomas in contact with or invading venous sinuses (hyperintense) [13]. T2-weighted sequences offer good contrast resolution for differentiating meningiomas from adjacent fibrous structures (like dura mater) or blood inside vessels with an inherent “black blood” effect. The added value of PET imaging remains unclear. It can add information and contribute to the identification of active tumor remnants, but it cannot replace fusion MRI/CT due to its lower spatial resolution [14].

Our definition of GTV is mainly consistent with definitions in literature. Randomized trials mainly report adjuvant treatment of high-grade meningiomas. The RTOG-0539 trial defined the GTV as the nodular enhancement and postoperative cavity, including hyperostosis and directly invaded bone [15]. This definition, with the addition of the dural tail, was also used in the ROAM/EORTC-1308 trial [16]. The dural tail is a reactive process that occurs due to direct tumor invasion or vascular congestion and edema [17]. The largest study of 179 patients with resected dural tails from convexity meningiomas found that 88.3% contained tumor cells (of which 95% were within 2.5 cm of the tumor base), without any differences between low- and high-grade tumors [18].

The main difference between the GTV definition in our study and those in literature [15, 16, 19–22] relates to the definition of the postoperative cavity, which, according to our experts, should be part of the CTV. Indeed, according to our experts, the tumor bed corresponds to microscopic rather than macroscopic disease, except for the postoperative residual tumor, which should be included in the GTV. While poRT is generally not required for grade I meningiomas, if it is indicated, partial or total omission of the surgical bed should be discussed [23].

Our recommendations suggest smaller margins than other major studies. CTV margins evolved from 10 to 40 mm in the three-dimensional RT (3DRT) era to 3–10 mm in the IMRT era, without decreasing 5-year local control rates (92–100% with 3DRT, and 93–97% with IMRT) [21, 24–26]. This margin reduction might be explained by improved diagnostic and therapeutic imaging providing better definitions of tumor extension.

The long-term survival of patients with grade I meningiomas and excellent late local control rates (93% at 5 years and 83–97% at 10 years) encourage smaller target volumes to be set, to minimize long-term toxicities [24, 25, 27–29]. This is consistent with the ESTRO-ACROP recommendations that do not suggest adding CTV margins to GTVs [6].

For grade II meningiomas, NCCN 2023 guidelines recommend a large CTV margin of 0.5 to 2 cm around the GTV and surgical bed and give no further details. The guidelines only recommend limiting margin expansion into the brain if there is no evidence of parenchymal invasion [7]. The RTOG-0539 and ROAM/EORTC-1308 trials recommend expanding the GTV by 10 mm to create the CTV, with the possibility of reducing it to 5 mm around natural barriers (without defining them) [15, 16]. Here, our experts recommend a smaller CTV margin of 5 mm. Press et al., in their series treating mostly grade II meningiomas, used a 5 mm expansion margin beyond the GTV, and 2-year and 5-year local control rates were 95 and 65%, respectively, similar to other results seen in literature [21, 22, 30]. In the series of Adeberg et al., using margins of 15–20 mm did not seem to improve local control rates [20]. Grade II meningiomas with a high Ki-67 index, with cutoff values between 2 and 20%, had worse overall (OS) and progression-free survival (PFS) [31]. These results suggest delineating meningiomas with a high Ki-67 index as grade III depending on other prognostic factors.

ESTRO-ACROP recommendations suggest a margin of 1 to 2 cm around the GTV, without distinguishing between grade II and III tumors [6]. With the objective of reducing margins to clinically relevant target volumes, our experts proposed a margin of 10 mm for grade III tumors. Few studies report on the mapping of recurrences. However, the majority appear to be in-field or out-field, with marginal recurrences occurring rarely [20, 32, 33]. For this aggressive disease, dose escalation rather than greater margins should be considered [34].

Cerebral venous sinuses correspond anatomically to a duplication of the dura mater with a vascular endothelium, contrasting with other venous and arterial structures [35]. In grade II and III tumors, our experts recommended including the sinus wall in the CTV when it is non-macroscopically invaded but in contact with the GTV. High-dose RT should be used with caution on venous sinuses, as thrombotic risks have been reported [36, 37].

The cortical bone represents a natural barrier, and appropriate imaging differentiates healthy and invaded bone [38]. In the absence of invasion, our experts did not recommend extending the CTV beyond the cortical bone. In poRT, conservation of the cranial bone flap for grade II or III tumors is being discussed within the neurosurgical community; the impact on OS or PFS is currently unknown [39]. Experts therefore did not recommend including the whole bone flap within the CTV but instead recommended including 5 or 10 mm within the CTV in the case of grade II or III tumors where the bone is invaded. The drill holes and osteotomy areas correspond to regions of rupture of the anatomical barrier. Surgeons should perform these in the periphery of the meningioma. If they come into contact with the CTV, our experts recommend including them. If a cranioplasty is performed, it should be also included in CTV.

Hyperostosis can relate to direct bone invasion by meningiomas [40], but reactive bone expansion is also possible. Postcontrast MRI sequences aid their discrimination [38]. Goyal et al. identified hyperostosis on preoperative imaging on CT and MRI in 75% of meningiomas. Tumor cells were present in the bone of 23.3% of patients who had hyperostosis before surgery [41]. Most of these tumors were grade I, suggesting that bone invasion is not in itself a sign of histologic aggressiveness. Therefore, the RTOG and EORTC trials recommended including hyperostosis in the GTV, and some retrospective series suggested a minimum margin of 3 mm around the GTV in cases of hyperostosis [15, 16, 26, 42]. Given the low relapse rate of grade I meningiomas, here our experts proposed not including hyperostosis in the GTV if high-quality MRI reveals no evidence of bone invasion but stated that hyperostosis must be included in the CTV for grade II and III tumors. Direct bone invasion must be included in the GTV regardless of the tumor grade.

Peritumoral brain edema is seen in approximately 60% of meningiomas without systematically correlating to tumor size. Aggressive meningiomas likely cause peritumoral edema by invading the brain [43]. Mantle et al. suggest that for every centimeter of peritumoral edema, the probability of brain invasion increases by 20% [44]. However, grade I meningiomas frequently cause peritumoral edema without brain invasion [43]. While the EORTC trials considered it to be part of the CTV for high-grade meningiomas, our experts did not recommend including it in its entirety; given the rarity of distant intraparenchymal, experts recommended including 5 mm of peritumoral edema in the CTV for grade II tumors and 10 mm for grade III tumors [16, 34, 45].

Recurrences along cranial or optic nerves are rarely described when the nerves are not invaded. Our experts recommended that where associated symptoms occur it should be assumed that cranial nerves are invaded, and therefore they should be included in the CTV. Optic nerves have the same embryological origin as the CNS; they are covered by meninges, unlike other cranial nerves, which are covered by perineurium [46]. For this reason, experts recommended that in the case of grade III tumors, cranial nerves should be included in the CTV if they contact the GTV. Optic nerve meningiomas, not discussed in this study, are the exception here; for these, a margin of 0 to 5 mm along the sheath is recommended [29, 47].

For SRT, all experts agreed with the recommendation that no additional margin from the GTV to the CTV is required. Many series have been published without additional margins and reported excellent local control rates, from 92 to 99% at 5 years [48–51]. The European Association of Neuro-Oncology (EANO) does not recommend the use of SRT for grade II and III tumors, outside of recurrence, although some studies describe similar results for small tumors [3, 52, 53].

## Conclusion

The current paper provides a detailed consensus proposal for delineating target volumes for meningioma radiotherapy, endorsed by a multidisciplinary group of brain cancer experts. These guidelines should be considered in the context of complete information about local disease staging, individual anatomical variations, and surgical procedures.

## Electronic supplementary material

Below is the link to the electronic supplementary material.

Supplementary Material 1

## Data Availability

The datasets used and/or analysed during the current study are available from the corresponding author on request.

## Abbreviations

ANOCEF: Association of French-speaking Neuro-oncologists
ACROP: Advisory Committee on Radiation Oncology Practice
CNS: Central nervous system
CTV: Clinical target volume
ESTRO: European Society for Radiation and Oncology
GTV: Gross tumor volume
IMRT: Intensity-modulated radiation therapy
IPRAS: Interpercentile range adjusted for symmetry
poRT: Postoperative radiotherapy
PTV: Planning Target Volume
RT: Radiotherapy
SRT: Stereotactic radiation therapy
WHO: World Health Organization
3DRT: Three-dimensional radiotherapy
