# Supplementary material for: Target volume delineation for radiotherapy of meningiomas: an ANOCEF consensus guideline
Source: Radiat Oncol. 2023 Jul 5;18:113. doi: 10.1186/s13014-023-02300-w (PMC10324255; doi:10.1186/s13014-023-02300-w)
Supplement: Supplementary file 1 — Supplementary Material 1 [file 13014_2023_2300_MOESM1_ESM.docx]

# APPENDIX

**Appendix 1:** Web questionnaire, with reformulated questions, answered by experts

For GTV and CTV, each proposal was scored from 1 (strongly disagree) to 9 (totally agree).

For CTV, additional margin to GTV was left as a free comment.

**Intensity modulated radiation therapy (IMRT)**

Imaging modality and sequences

1) Which imaging modality/sequence is required for the target volume delineation on the planning CT-Scan?

|  | Not recommended | Optional | Strongly recommended | Mandatory |
| --- | --- | --- | --- | --- |
| Planning CT-Scan without contrast |  |  |  |  |
| Planning CT-Scan with contrast |  |  |  |  |
| Fusion MRI without gadolinium injection in T1-weighted sequence |  |  |  |  |
| Fusion MRI with gadolinium injection in T1-weighted sequence |  |  |  |  |
| Fusion MRI with T2 weighted sequences in 1 or 3 planes |  |  |  |  |
| Fusion MRI FLAIR sequence |  |  |  |  |
| Fusion with TEP 68Ga-DOTA |  |  |  |  |
| Fusion MRI sequences with cancellation of the blood signal by spin echo method ("Black blood") |  |  |  |  |
| Fusion MRI with High spatial Resolution (HR) sequences |  |  |  |  |
| Fusion with pre-operative MRI (in case of post-operative radiotherapy) |  |  |  |  |

IMRT: Which structures have to be included in the gross tumor volume (GTV)?

*Grade I Meningioma: exclusive or post-operative radiotherapy*

2) Nodular dural enhancement

3) Thickened meninges (if present)

4) Directly invaded bone (if present)

*Grade II Meningioma: exclusive or post-operative radiotherapy*

5) Nodular enhancement

6) Thickened meninges (if present)

7) Directly invaded bone (if present)

*Grade III Meningioma: exclusive or post-operative radiotherapy*

8) Nodular enhancement

9) Thickened meninges (if present)

10) Directly invaded bone (if present)

*Tumor bed definition*

11) Tumor bed belongs to the CTV rather than the GTV?

12) Tumor bed is defined by the strict operative cavity (defined 3-5 weeks after surgery) + ... mm of cerebral parenchyma.

*Tumor bed delineation*

13) Grade I: Tumor bed needs to be included in target volume (GTV or CTV).

14) Grade II: Tumor bed needs to be included in target volume (GTV or CTV).

15) Grade III: Tumor bed needs to be included in target volume (GTV or CTV).

IMRT: What margin is used to define the clinical target volume (CTV)?

*Grade I Meningioma: exclusive or post-operative radiotherapy*

16) CTV include margin in normal brain tissue around nodular enhancement.

17) CTV include margin in normal brain tissue around the tumor bed, as previously defined (if present).

18) CTV include margin along normal meninges (not thickened).

19) CTV include margin in healthy bone.

20) CTV include hyperostosis.

21) CTV include whole peritumoral oedema.

22) CTV include cranial flap.

23) CTV include venous sinuses in contact with the GTV.

24) CTV include other vascular structures (artery) in contact with the GTV.

25) CTV include the optic nerve in contact with the GTV.

26) CTV include other nerve structures (cranial nerves) in contact with the GTV.

*Grade II Meningioma: exclusive or post-operative radiotherapy*

27) CTV include margin in normal brain tissue around nodular enhancement.

28) CTV include margin in normal brain tissue around the tumor bed, as previously defined (if present).

29) CTV include margin along normal meninges (not thickened).

30) CTV include margin in healthy bone.

31) CTV include hyperostosis.

32) CTV include whole peritumoral oedema.

33) CTV include cranial flap.

34) CTV include venous sinuses in contact with the GTV.

35) CTV include other vascular structures (artery) in contact with the GTV.

36) CTV include the optic nerve in contact with the GTV.

37) CTV include other nerve structures (cranial nerves) in contact with the GTV.

*Grade III Meningioma: exclusive or post-operative radiotherapy*

38) CTV include margin in normal brain tissue around nodular enhancement

39) CTV include margin in normal brain tissue around the tumor bed, as previously defined (if present)

40) CTV include margin along normal meninges (not thickened).

41) CTV include margin in healthy bone.

42) CTV include hyperostosis.

43) CTV include whole peritumoral oedema.

44) CTV include cranial flap.

45) CTV include venous sinuses in contact with the GTV.

46) CTV include other vascular structures (artery) in contact with the GTV.

47) CTV include the optic nerve in contact with the GTV.

48) CTV include other nerve structures (cranial nerves) in contact with the GTV.

*Meningioma exclusive or post-operative radiotherapy: Regardless of grade*

49) CTV margin needs to be increased for rapidly progressive meningioma during follow-up before radiotherapy.

50) CTV margin needs to be increased for recurrent meningioma (without previous radiotherapy).

*When histological grade is not available, which factors are relevant to delineate target volume as a high grade (II-III) meningioma?*

51) Young age (< 60 years)

52) Non-menopausal status

53) Skull base location

54) Cranial vault location

55) Large size meningioma

56) Recurrent meningioma

57) Rapidly progressive meningioma

58) Hyperostosis

59) Bone invasion

60) Necrosis

61) Irregular contours (lobulated)

62) Peritumoral oedema

63) Significant cerebral oedema for a moderate size lesion

64) Aggressive criteria on MRI (High-intensity on T2-weighted sequences, low apparent diffusion coefficient, highly increased perfusion, ...)

65) None. When histological grade is not available, I delineate as a low grade.

**Stereotactic radiation therapy (SRT)**

Imaging modality and sequences

66) Which imaging modality/sequence is required for the target volume delineation on the planning CT-Scan?

|  | Not recommended | Optional | Strongly recommended | Mandatory |
| --- | --- | --- | --- | --- |
| Planning CT-Scan without contrast |  |  |  |  |
| Planning CT-Scan with contrast |  |  |  |  |
| Fusion MRI without gadolinium injection in T1-weighted sequence |  |  |  |  |
| Fusion MRI with gadolinium injection in T1-weighted sequence |  |  |  |  |
| Fusion MRI with T2 weighted sequences in 1 or 3 planes |  |  |  |  |
| Fusion MRI FLAIR sequence |  |  |  |  |
| Fusion with TEP 68Ga-DOTA |  |  |  |  |
| Fusion MRI sequences with cancellation of the blood signal by spin echo method ("Black blood") |  |  |  |  |
| Fusion MRI with High spatial Resolution (HR) sequences |  |  |  |  |
| Fusion with pre-operative MRI (in case of post-operative radiotherapy) |  |  |  |  |

Indications

67) SRT is suitable for high-grade (grade II-III) meningiomas (excluding re-irradiation).

SRT: Which structures have to be included in the gross tumor volume (GTV), regardless of grade?

68) Nodular enhancement

69) Thickened meninges (if present)

70) Directly invaded bone (if present)

Tumor bed delineation

71) Tumor bed, as previously defined, needs to be included in target volume (GTV or CTV).

SRT: What margin is used to define the clinical target volume (CTV), regardless of grade?

72) CTV include margin in normal brain tissue around nodular enhancement.

73) CTV include margin in normal brain tissue around the tumor bed, as previously defined (if present).

74) CTV include margin along normal meninges (not thickened).

75) CTV include margin in healthy bone.

76) CTV include hyperostosis.

77) CTV include peritumoral oedema.

78) CTV include cranial flap.

79) CTV include veinous sinuses in contact with the GTV.

80) CTV include other vascular structures (artery) in contact with the GTV.

81) CTV include the optic nerve in contact with the GTV.

82) CTV include other nerves structures (cranial nerves) in contact with the GTV.

SRT: How to adapt volumes according to the evolving tumor profile?

83) CTV margin needs to be increased for rapidly progressive meningioma during follow-up before radiotherapy.

84) CTV margin needs to be increased for recurrent meningioma (without previous radiotherapy).
